# Understanding the Health and Social Determinants of Health Needs of Resettled Afghan Refugees in Houston, Texas

**DOI:** 10.1177/2752535X251361011

**Published:** 2025-07-23

**Authors:** Karissa Chesky, Angelica Garcia, Aaron Pathak, Imran Humza Hanif, Srijana Shrestha, Sophia Banu

**Affiliations:** 1 3989 Baylor College of Medicine, Houston, TX, USA; 2 14736 Texas A&M University, College Station, TX, USA; 3 6097 Wheaton College, Norton, MA, USA; 4 Menninger Department of Psychiatry and Behavioral Sciences, 333210 Baylor College of Medicine, Houston, TX, USA

**Keywords:** refugee, resettlement, migration, public health, health equity, health disparities

## Abstract

**Purpose:**

The global refugee crisis includes Afghan refugees, driven by decades of conflict. Understanding the currently unidentified, unique challenges of this group upon resettlement in the United States is crucial for bettering health outcomes. This needs assessment identifies the challenges surrounding health experiences of resettled Afghan refugees in Houston, Texas.

**Methods:**

Adult Afghan refugees resettled in Houston, Texas were surveyed via a needs assessment adapted from validated health screeners (PRAPARE, the RHS15, and CoPaQ) with translator assistance. Health experiences across demographics, urgent needs, accessibility, and healthcare services were assessed.

**Results:** 73 participants were surveyed (median age: 33 years, 74% female). Most had lived in the U.S. for 1–3 years, primarily spoke Dari, lacked English proficiency, were unemployed, and earned less than $20,000 annually. Top needs were employment, food, and transportation, and key accessibility issues included transportation, clothing, and learning English. Though many had health insurance, only some felt comfortable visiting a doctor alone and felt understood by their physician. About one-third rated their health as fair or poor. For medical visits, most relied on case managers and interpreters for navigating appointments, traveled by car, and had wait times under an hour. Social determinants like housing, childcare, and healthcare access showed significant variation.

**Conclusion:**

Findings reveal key elements, including language, transportation, provider communication, that shape the health experiences of resettled Afghan refugees. These access contributors can inform more responsive healthcare systems. Given the urgency of our analysis, healthcare, governmental and community programs should pursue targeted approaches to meet this population's needs.

## Introduction

According to the United Nations High Commissioner for Refugees (UNHCR), over 122.6 million individuals are forcibly displaced from their homes, creating an unprecedented global refugee crisis.^1^ Of this group, an estimated 37.9 million are refugees, often denied basic rights such as nationality, access to healthcare, education, employment, and the liberty of movement and migration. Despite people fleeing persecution and conflict for thousands of years, it was not until the wake of World War II that the UNHCR was created to help serve, relocate, and grant asylum to displaced Europeans, who were primarily Jewish refugees of the Holocaust, later expanding to the entire global refugee community.^2^ Most refugees receive support in the country to which they fled, referred to as the second country, until they can safely return home. A minority of them will be granted citizenship in the second country; even fewer are resettled in a third country. Although only one percent of all refugees will eventually be resettled in a third country, the United States is one of the world’s largest resettlement recipients, historically welcoming approximately two thirds of this small population, though current political climates in the United States threaten this reciprocity.^3^

Afghan refugees are a prominent group of the international refugee population at large. The Afghan people have faced over four decades of conflict and instability, steadily leading up to the Taliban’s takeover of Kabul in 2021, lending to further hardship.^4^ Notably, the United States has had a two-decade military involvement in Afghanistan, triggered by the September 11 attacks, to dismantle al-Qaeda and then carry out a counterinsurgency strategy to rebuild the country.^5^ These factors have driven to a growing 6.4 million Afghan refugees globally as of 2023, amounting to one of the largest refugee crises worldwide.^6^ In the United States, the number of Afghan refugees nearly quadrupled in number between 2010 and 2022 from an estimated 54,000 to 195,000, according to Migration Policy Institute (MPI), in tandem with the humanitarian crises in Afghanistan throughout this period and the moral responsibilities that the United States had made to Afghans impacted by war.^7^ Amongst this effort, Houston has also become a significant location for Afghan resettlement. It is estimated that between 5000 and 15,000 Afghan refugees have settled in Houston, making it one of the largest resettlement areas for this population in the United States.^8^ This wide range may reflect limitations in tracking secondary migration, attracting Afghan refugees who were initially resettled in other parts of the United States but are seeking more developed refugee communities that have already been established.

Houston’s prominence as an effective center for refugee resettlement, particularly within the broader political climate surrounding immigration in Texas, is notable. Houston has long embodied this role, especially since the influx of Vietnamese refugees in the 1970s following the collapse of the Saigon regime.^9^ Today, the greater Houston area remains as one of the most diverse and refugee-strong regions in the entire United States, hosting an estimated 1.7 million immigrants as of 2023, equating to one-quarter of the entire population in the nine-county metro area.^10^ While Houston continues to be recognized for its multiculturalism and openness to refugees, it exists within a state with a legislative environment that grows increasingly hostile towards migrants. For example, Operation Lone Star, a border security initiative launched in 2021, has led to the arrest and criminal charges against a growing total of hundreds of thousands of migrants.^11^ Senate Bill 4 was also passed in 2023, allowing Texas police to detain individuals that they believe to have entered Texas without legal immigration status.^12^ Despite these legislative changes, Houston remains vocal in opposition, with one of the nation’s longest urban area studies demonstrating that most residents point to its diversity, migrant population, and economic opportunity with pride.^13^ This contrast between state lawmakers and the Houston community highlights the complex dynamics of refugee settlement in Texas and points towards local efforts that continue to foster acceptance towards the refugee community.

Upon arrival to the United States, Afghan refugees encounter a host of new experiences, including navigation of complex healthcare systems, securing employment, and adapting to a new cultural and linguistic environment.^14^ Within the healthcare system specifically, there are various resources to support refugees, including financial benefits at federal, state, and local levels. Coverage options include federally funded Medicaid, the Children’s Health Insurance Program, the Affordable Care Act, and Medicare, with varying levels of expansion at the state level.^15^ The Refugee Medical Assistance program provides short-term medical coverage to refugees, while the Refugee Medical Screening program provides a comprehensive health assessment to newly arrived refugees, both federally organized.^15^ While these resources are in place to support refugee health, difficulties remain, putting Afghan refugees at an increased risk for poorer health outcomes than the general population.^16^ Elements that contribute to said challenges include those such as cultural factors that have been shown to play a critical role in the health outcomes of Afghan refugees. Afghan culture places a strong emphasis on community, prayer, and resilience, which can positively impact physical and mental health treatments. However, the lack of cultural literacy among healthcare providers often results in lower-quality care for Afghan refugees.^17^ Additionally, it has been demonstrated Afghan refugees can face faith- and attire-related discrimination, a lack of native-language speaking staff, and a general sense of alienation within the healthcare system.^18^ This cultural disconnect can lead to misunderstandings and mistrust between refugees and healthcare providers, further exacerbating health disparities despite healthcare coverage options.

To combat these challenges, resettlement programs have been established to support refugees in their transition to the United States. The overarching goal of resettlement programs is to deliver durable solutions for resettled refugees by ensuring protection and access to the civil, political, economic, social and cultural rights granted to nationals, while providing them opportunities and support throughout the integration process.^19^ Historically, resettlement agencies function at the national, state and local affiliate level. Following registration with the UNHCR and then referral for resettlement to the United States, the Bureau of Population, Refugees and Migration serves as the primary governmental entity to coordinate on behalf of a refugee with domestic resettlement agencies. Such domestic agencies largely fall within a group of 10, largely faith-based organizations, that handle the logistics of refugee resettlement by working closely with multiple local partners to prepare for their arrival. Arranged services typically include reception, placement, cultural orientation, health and social services, employment, language learning, transportation, and more. While these programs provide benefit, there is a large variation of each region’s own nuances, creating a wide variability in services, needs, barriers, and resources within the same country.^20^ These agencies are also frequently overwhelmed by the volume of refugees, leading to insufficient support and forcing many refugees to independently navigate their new environment. To identify what the unique needs of a refugee population in a specific area are, a needs assessment can be performed. In general, a needs assessment identifies local needs and resources in hopes of enabling changemakers to improve and serve their community in the most logical and efficient manner possible.^21^ A combination of information gathering, community engagement, critical analysis, and a focused action plan are all staple components of creating an effective needs assessment. Furthermore, understanding specific need gaps before jumping to solutions can increase the likelihood of success for future established endeavors, and haphazard interventions can often lead to unintended, harmful consequences or a waste of resources.

A comprehensive understanding of the specific health and social needs of Afghan refugees is essential for improving their overall well-being. Generally, there is a need for a more tailored approach to healthcare for Afghan refugees, but there is limited analysis of the health-related needs of this population to date. Given the clear lack of understanding of this population’s needs, in the setting of Houston, Texas where Afghan refugees comprise a large and important part of the community, this needs assessment study was conducted. The aim of this needs assessment is to describe the general health and related social needs of Afghan refugees in Houston, Texas. By identifying these needs and the challenges they face, this study seeks to inform healthcare providers, students in related fields, community members, and governmental agencies on the most effective measures to address these issues. Ultimately, this research aims to improve health outcomes and support the successful integration of Afghan refugees into the local community.

## Methods

We conducted a needs assessment of adult Afghan refugees resettled in Houston, Texas. This study was conducted in accordance with the Declaration of Helsinki and was approved by the Baylor College of Medicine Institutional Review Board (Protocol H-43090). The study participants were recruited in collaboration with The Alliance (formerly known as The Alliance for Multicultural Community Services), a local refugee resettlement agency. There are other resettled agencies in Houston, including but not limited to YMCA International Services, Interfaith Ministries for Greater Houston, and Catholic Charities. These programs work in unison with each other and community partners to promote self-sufficiency in some of the most vulnerable members of the Houston community. Inclusion criteria for the study included resettled refugee adult clients between the ages of 18–64 years of The Alliance whose primary language is either Farsi, Pashto, or a Farsi-derived dialect, and who were resettled between 2016 and 2024 (including during the COVID-19 pandemic). Clients of The Alliance whose primary language is not Farsi, Pashto, or a Farsi-derived dialect, or who were resettled in the United States before 2016, were excluded from the study.

The needs assessment for this study was created through collaboration with community partners and by examining needs assessment questionnaires that have been used in other refugee populations through literature review. In efforts to maintain collaboration and community engagement, the content of this survey was informed by a previous qualitative investigation, in which interviews were conducted with staff members of the resettlement agencies and leaders of the various refugee communities. The majority of questions utilized in the survey were then borrowed or adapted from validated screeners such as PRAPARE, the RHS15, and CoPaQ.^22–24^

Investigators recruited clients that met inclusion criteria to be study participants through the help of an Alliance case manager during their routine scheduled appointments. The researchers made clear to the clients that the services they receive from the Alliance are in no way contingent on their participation in the study. If the client was interested in participating in the study, a trained staff member from our team conducted an oral orientation about the purpose of the study and the study procedures. A case manager served as a translator and interpreter. It was emphasized especially that the study is voluntary to the participant, and that the participant may choose to conclude their participation in the study at any time and without negative consequences. Any client enrolled in the study and therefore participated in the 1-h needs assessment survey session received compensation in the form of food items, such as rice, flour, sugar, tea, peanut butter, etc., valued at no more than $10 in total. Any case managers acting as a translator and interpreter for the duration of the assessment (ranging from 3 hrs to 5 hrs) of survey sessions received compensation of $50 for their time.

### Measures

The 78-item questionnaire was administered between September 2021–December 2023. Demographic information of the participants included: age, country of origin, country of nationality, country of birth, gender, marital status, languages spoken, primary language spoken in the home, ability to read in native language, ability to write in native language, length of time in the United States (at time of survey), housing status, household yearly income, how many adults in the household, are any adults in the household disabled, how many children (17 and younger) are in the household, are any children in the household disabled, currently pregnant (if yes, was the pregnancy planned), employment status (if yes, remote or in-person, number of jobs, how many hours of work per week; if no, reasons for unemployment), if looking for more work, highest level of completed education (if degree earned, what country was the degree obtained), current education enrollment status (yes, no and do not want to enroll, no but would like to enroll), disability benefits, and welfare benefits. Additional questions inquired about health experiences under the following domains: general needs, affordability, scheduling, transit, communication, providers, and healthcare barriers.

### Data Analysis

Descriptive analysis was conducted to characterize four main categories: demographics, needs, accessibility via social determinants of health, and healthcare services. R version 4.3.1 (R Foundation for Statistical Computing, Vienna, Austria) was used for statistical analysis.

## Results

### Demographics

Table 1 presents the participant demographics. 73 participants agreed to participate in the study and were surveyed. The median age of the participants was 33 years (IQR: 30–38), 74% female, 26% male. Nearly all participants reported Afghanistan as their country of origin. Most participants were born in Afghanistan (91.8%) and reported Afghanistan as their nationality (84.9%). The majority (47.9%) have been in the United States between 1 and 3 years, 17.8% have for 3–8 months, and 15.1% for less than 3 months. Most (57.5%) reported Dari (Farsi-derived dialect) as their primary language spoken at home, followed by Pashto (12.3%) and Farsi (9.6%). The majority could read (79.5%) and write (67.1%) in their native language. Most (57.5%) reported no English proficiency. The majority (93.2%) have their own apartment or house, while 5.5% live with others or friends. Most (34.2%) earn less than $10,000, 24.7% between $10,000–$20,000, and 20.5% between $20,000–$30,000. Only 2.7% earn more than $30,000. The median number of adults living together is 1 (IQR: 1–2), and children 3 (IQR: 2–5). 4.1% report having disabled adults or children in the household. Most (72.6%) were not pregnant, 24.7% did not respond. The majority are not employed (65.8%). 17.8% have no formal education, 26% have less than a high school education, 23.3% have a high school diploma or GED. Most (37%) want to enroll into school, while 30.1% do not want to enroll and 6.4% are currently enrolled.

**Table 1.** Demographics.

Characteristics	Category	*n* (%)
Age (median [IQR])		33.00 [30.00, 38.00]
Country of origin	Afghanistan	72 (98.6)
	No response	1 (1.4)
Nationality	Afghanistan	62 (84.9)
	Pashtun	3 (4.1)
	Tagik	3 (4.1)
	Persian	1 (1.4)
	No response	4 (5.5)
Birth country	Afghanistan	67 (91.8)
	Iran	1 (1.4)
	Pakistan	1 (1.4)
	No response	4 (5.5)
Gender	Female	54 (74.0)
	Male	19 (26.0)
Marital	Married	62 (84.9)
	Single	7 (9.6)
	Widow	1 (1.4)
	Widower	1 (1.4)
	No response	2 (2.7)
Home language spoken	Dari	42 (57.5)
	Pashto	9 (12.3)
	Farsi	7 (9.6)
	Shughnani	1 (1.4)
	Turkish	1 (1.4)
	English	1 (1.4)
	No response	12 (16.4)
Ability to read in native language	Yes	58 (79.5)
	No	15 (20.5)
Ability to write in native language	Yes	49 (67.1)
	No	17 (23.3)
	No response	7 (9.6)
Self-reported English literacy	Yes	28 (38.4)
	No	42 (57.5)
	No response	3 (4.1)
Time in the USA	<3 months	11 (15.1)
	3 months–8 months	13 (17.8)
	9 months–1 year	4 (5.5)
	1–3 Years	35 (47.9)
	4 years–5 years	7 (9.6)
	>5 years	2 (2.7)
	No response	1 (1.4)
Housing	Yes, I have my own apartment/house	68 (93.2)
	No, I live with others/friends	4 (5.5)
	No response	1 (1.4)
Household income	<$10,000	25 (34.2)
	$10,000–20,000	18 (24.7)
	$20,000–30,000	15 (20.5)
	>$30,000	2 (2.7)
	No response	13 (17.8)
Adults that live with participant (median [IQR])		1.00 [1.00, 2.00]
Disabled adults that live with participant	Yes	3 (4.1)
	No	39 (53.4)
	No response	31 (42.5)
Children that live with participant (median [IQR])		3.00 [2.00, 5.00]
Disabled children that live with participant	Yes	3 (4.1)
	No	59 (80.8)
	No response	11 (15.1)
Pregnant	Yes	2 (2.7)
	No	53 (72.6)
	No response	18 (24.7)
Employment	Yes	15 (20.5)
	No	48 (65.8)
	No response	10 (13.7)
Education	No formal education	13 (17.8)
	Less than high school	19 (26.0)
	High school or GED	17 (23.3)
	University (bachelor’s)	11 (15.1)
	Graduate (master’s or doctorate)	2 (2.7)
	No response	11 (15.1)
Current education status	Yes	12 (16.4)
	No, but would like to enroll	27 (37.0)
	No, and do not want to enroll	22 (30.1)
	No response	12 (16.4)

### Top Three Needs

Table 2 presents the top three needs. Employment (14.4%), food (13.7%), and transportation (12.3%) were the most frequently listed as the top three needs by participants. Other common needs included housing concerns (rent, utilities payment, understanding utilities, furniture, appliances), healthcare, learning English and education, finances, clothing, childcare, and budgeting. 6.8% of participants reported “everything” as a top three need.

**Table 2.** Self-Reported Top Three Needs.

Needs	*n*	%
Employment	21	14.4
Food	20	13.7
Transportation	18	12.3
Rent	13	8.9
Healthcare	10	6.8
Everything	10	6.8
Learning English	9	6.2
Finances	8	5.5
Utilities payment	6	4.1
Understanding utilities	5	3.4
Clothing	4	2.7
Childcare	4	2.7
Budgeting	4	2.7
Furniture	2	1.4
Reuniting family	1	0.7
Education	1	0.7
Appliances	1	0.7
No response	9	6.2

### Social Determinants of Health

Table 3 reports social determinants of health, or the nonmedical factors that impact health outcomes. The majority of participants reported “yes” for having accessibility troubles for clothing (52.1%), transportation (57.5%), and learning English (57.5%). The majority of participants reported “no” for having accessibility troubles for food (46.6%), utilities (45.2%), healthcare (56.2%), phone access (52.1%), and childcare (47.9%). 37% of participants received welfare, 41.1% did not. 74% of participants did not receive disability benefits, 24.7% did not respond.

**Table 3.** Social Determinants of Health Measures.

Measures	Response	*n* (%)
Receives disability	Yes	1 (1.4)
	No	54 (74.0)
	No response	18 (24.7)
Receives welfare	Yes	27 (37.0)
	No	30 (41.1)
	No response	16 (21.9)
Food troubles	Yes	31 (42.5)
	No	34 (46.6)
	No response	8 (11.0)
Utilities troubles	Yes	30 (41.1)
	No	33 (45.2)
	No response	10 (13.7)
Healthcare troubles	Yes	23 (31.5)
	No	41 (56.2)
	No response	9 (12.3)
Phone troubles	Yes	28 (38.4)
	No	38 (52.1)
	No response	7 (9.6)
Clothing troubles	Yes	38 (52.1)
	No	29 (39.7)
	No response	6 (8.2)
Childcare troubles	Yes	25 (34.2)
	No	35 (47.9)
	No response	13 (17.8)
Transportation troubles	Yes	42 (57.5)
	No	23 (31.5)
	No response	8 (11.0)
English troubles	Yes	42 (57.5)
	No	21 (28.8)
	No response	10 (13.7)

### Healthcare

Table 4 reports healthcare service-related data. 65.8% reported having health insurance, 17.8% reported not having health insurance, and 16.4% did not respond. For those with insurance, 60.3% reported having Medicare/Medicaid. Most participants rated their overall health as “very good” (20.5%), followed by “good” (19.2%), “fair” (17.8%), poor (11%) and excellent (11%). The majority of participants had a case manager (32.9%) to schedule medical appointments, and most reporting <15 min (34.2%) for time taken to schedule such appointments. Most traveled to their medical appointments by car (57.5%), followed by bus (16.4%), and walking (6.8%). Wait time for medical appointments was 15–30 min for most (26%), followed by 30 min–1 h (23.3%), and >1 h (20.5%). There was an interpreter available for most participants at their medical appointments (72.6%); 69.6% of those that had an interpreter available used it, with the majority of interpretation services being via phone (57.5%). Most participants’ appointment time was 15–30 min (39.7%). 60.3% felt comfortable going alone to their doctor, 24.7% did not feel comfortable, and 15.1% did not respond. 68.5% felt understood by their doctor, 15.1% did not, and 16.4% did not respond. 74% would return to their doctor, 12.3% would not, and 13.7% did not respond. Most reported having a place to go to when they are sick (65.8%) and for when their children are sick (65.8%).

**Table 4.** Healthcare Services Measures.

Measures	Response	*n* (%)
Health insurance	Yes	48 (65.8)
	No	13 (17.8)
	No response	12 (16.4)
Overall health rating	Excellent	8 (11.0)
	Very good	15 (20.5)
	Good	14 (19.2)
	Fair	13 (17.8)
	Poor	8 (11.0)
	No response	15 (20.5)
Insurance type	Medicare/Medicaid	44 (60.3)
	Other	5 (6.8)
	No response	24 (32.9)
Who schedules doctor appointments	Case manager	24 (32.9)
	Self	21 (28.8)
	Family	17 (23.3)
	Friend	5 (6.8)
	No response	6 (8.2)
Time taken to schedule doctor appointments	<15 min	25 (34.2)
	15–30 min	21 (28.8)
	30 min–1 h	3 (4.1)
	>1 h	6 (8.2)
	No response	18 (24.7)
Time to transport to doctor appointments	<15 min	11 (15.1)
	15–30 min	31 (42.5)
	30 min–1 h	16 (21.9)
	>1 h	3 (4.1)
	No response	12 (16.4)
Transport method to doctor appointments	Car	42 (57.5)
	Bus	12 (16.4)
	Walk	5 (6.8)
	Other	2 (2.7)
	No response	12 (16.4)
Wait time	<15 min	8 (11.0)
	15–30 min	19 (26.0)
	30 min–1 h	17 (23.3)
	>1 h	15 (20.5)
	No response	14 (19.2)
Interpreter available	Yes	53 (72.6)
	No	4 (5.5)
	No response	16 (21.9)
Used interpreter	Yes	51 (69.9)
	No, and did not need it	11 (15.1)
	No response	11 (15.1)
Interpreter type	Phone	42 (57.5)
	In person	8 (11.0)
	Both	2 (2.7)
	Not applicable	11 (15.1)
	No response	10 (13.7)
Comfortable going alone to their doctor	Yes	44 (60.3)
	No	18 (24.7)
	No response	11 (15.1)
Felt understood by their doctor	Yes	50 (68.5)
	No	11 (15.1)
	No response	12 (16.4)
Would return to their doctor	Yes	54 (74.0)
	No	9 (12.3)
	No response	10 (13.7)
Appointment time	<15 min	5 (6.8)
	15–30 min	29 (39.7)
	30–60 min	9 (12.3)
	>1 h	17 (23.3)
	No response	13 (17.8)
Place to go when sick	Yes	48 (65.8)
	No	16 (21.9)
	No response	9 (12.3)
Place to take children when sick	Yes	48 (65.8)
	No	11 (15.1)
	No response	14 (19.2)

## Discussion

The results of this needs assessment reveal that Afghan refugees face vulnerabilities when resettling to Houston, Texas across several areas. Our demographic and social determinants of health data alone highlight the complexities of this population. The participant cohort is female predominant (74%) with a median age of 33 years. Because the study methodology lent to inclusion of participants who were more available due to lack of employment obligations, these cohort characteristics reflect a younger population that follows a patriarchal structure common in some refugee groups and frequently associated with women bearing the stressors of primary household and child caretaking.^25^ Most participants spoke Dari as their primary language (57.5%), but not all participants could read and write in their native language. Moreover, greater than half of participants reported limited proficiency in English (57.5%). Here, linguistic barriers to the English language, but also to general literacy itself, become apparent. Education emerges tangentially as an area of need, as 17.8% have no formal education and 26% have less than a high school education, yet 37% of participants express a desire to enroll in school. This combination of low literacy and education levels suggests that there is a severe limitation to participants’ ability to wholly navigate their community, including the healthcare system and understanding their health at large.^26^ However, the significant desire of participants seeking educational opportunities may reflect an awareness within the cohort that formal education is necessary to increase accessibility to employment, economic, and overall quality of life in the regional societal structure. This sentiment is consistent with previous demonstrations that female Afghan refugees specifically appear to be motivated in developing English proficiency, but have had difficulty doing so due to pedagogical barriers, cultural expectations of Afghan women that take time away from formal education, and psycho-emotional issues including self-esteem, dedication, and age.^27^

A large proportion of participants been resettled in the United States for 1–3 years (47.9%). Despite this period, only 20.5% of participants reported being employed and most reported household incomes below $20,000, with 34.2% of participants earning less than $10,000. Additionally, housing conditions, while generally stable (93.2% reported having their own apartment or home), may still be a cause of concern given the reported low-income levels. Most families reported living in households with a median of one adult and three children, comparable to the 2024 Central Intelligence Agency statistic of women in Afghanistan having an average fertility rate of 4.43, further emphasizing a financial strain many experience when supporting larger families on limited incomes.^28^ These demographic factors all point to significant economic hardship, reflected in their top three reported needs: employment (14.4%), food (13.7%), and transportation (12.3%). Notably, 6.8% of participants reported “everything” as a top three need, disclosing a burdening sense of need that exceeds specific categories. The demonstration of these needs parallel previously identified findings of needs of the general resettled refugee population in Houston, reiterating their strong influence.^29^ The high number of participants having difficulty accessing basic needs indicates an urgent necessity for more robust support systems aimed at improving specifically education and economic stability for this group.

Healthcare accessibility presented mixed results. 65.8% reported having health insurance, 17.8% reported not having health insurance, and 16.4% did not respond. While several participants did report having health insurance, largely through Medicare or Medicaid (60.3%), a significant portion of all participants rated their health as only “fair” (17.8%) or “poor” (28.8%). Contributors to these insufficient health outcomes may parallel the demonstrated difficulty of resettled refugees in the United States with insurance coverage seeing any specialty service beyond their covered primary healthcare services.^30^ Other factors towards these health ratings may stem from inefficiencies in participants’ healthcare service delivery. Specifically, most participants relied on their case manager to serve as their primary mechanism for making medical appointments (32.9%). Although efficient in the short-term, with most reporting <15 min (34.2%) for time taken to schedule such appointments, reliance on a third party may lend to healthcare underutilization, a limited ability to more freely contact their healthcare provider, an incomplete understanding of appointment information, and slowed personal growth in how to independently navigate the healthcare system. Participants traveled to their medical appointments using both private and public transportation systems, including via car (57.5%), via bus (16.4%), and walking (6.8%). Several participants reported waiting 30 min–1 h for their medical appointments (23.3%), and about one-fifth had to wait for more than 1 h (20.5%). Notably, 72.6% had access to interpreters, although 69.9% relied on phone-based interpretation, likely limiting comprehensive communication with healthcare providers as face-to-face and video interpretation are typically preferred modalities.^31^ Despite these challenges, many participants expressed that they were comfortable going alone to their doctor (60.3%), felt understood by their doctor (68.5%), and a willingness to return to their doctors (74%), indicating a degree of satisfaction with their care. These effective attributes of this cohort’s experiences with their doctor contradict those previously reported in other refugee populations, including fear for losing legal status, inability to file complaints, confusion about insurance coverage, and lack of personalized care.^32^ Consideration of the specific attributes of the medical provider that lent to positive aspects of the healthcare provider-relationship in this study, including but not limited to communication styles, language or cultural awareness, and interpersonal skills, should be more deeply investigated so that future interactions intentionally embody these effective attributes.^33^ It is also noteworthy that the study was conducted during the SARS-CoV-2 pandemic (COVID-19), adding a compelling layer to participants’ healthcare access. COVID-19 significantly disrupted healthcare systems globally and intensified barriers to health, especially for refugee communities, who face some of the highest levels of vulnerability at baseline.^34^ Reported impacts of COVID-19 on refugees, including economic hardship, disease vulnerability, mental illness exacerbations, and communication challenges, align with the findings of our cohorts’ resettlement experiences, that likely exacerbated existing systemic inequities.^34^

The study faced several limitations. The participant sample did not include children, and it exhibited gender and age biases as younger families with children were more likely to have relocated, thus excluding older individuals. There was also convenience sampling as random sampling was not a reasonable recruitment process for this study. The participants were primarily those under the management of a resettlement agency, contributing to both the convenience sampling and the likelihood that they had greater access to resources compared to refugees without such structured support. This assistance may have led to inflated scores, in addition to the possibility that some participants might have been hesitant to critically assess the American healthcare system when speaking to someone connected to that system. Additionally, one participant did not provide a response to the “country of origin” demographics question, most likely due to human error that led to survey recording being incomplete at the time of survey administration. The IRB protocol was also modified mid-study due to the COVID-19 pandemic to ensure that the target data was collected.

## Conclusions

This needs assessment demonstrates the healthcare and social determinant of health needs of resettled Afghan refugees in Houston, revealing their dynamic interplay. Addressing both domains is necessary to improve health outcomes of this group. Now, healthcare providers, government officials, community partners, learners, and the community at large can utilize these findings to see through the lens of resettled refugees and better guide their interactions with this population. Specifically, the identified areas where need prevails and where efforts have impacted positively should be addressed by these parties. For instance, our study has demonstrated that prioritization of efforts should be towards targeted educational programs for Afghan women who have exhibited a strong interest in learning opportunities, but also to broader, systemic investments in economic stability, healthcare, and education systems. The comfort that refugees report with healthcare providers additionally suggests a valuable model to be replicated for culturally sensitive relationships across other services. While many refugees access public health insurance, persistent low health outcomes highlight that quality, not just availability, of healthcare needs to be improved. Moving forward, this needs assessment can support further research of each nuanced subtopic uncovered here to lend towards a successful integration of such recommendations into healthcare practices, educational programs, and policymaking related to this population. Measuring the success of meeting their needs following implementation of various support programming is also necessary. This is particularly pertinent in the current political landscape, in which termination of Temporary Protected Status for over 11,000 individuals from Afghanistan who reside in the United States will occur in July 2025. This decision will put many resettled Afghan refugees and their loved ones at increased health, psychosocial, and general risk, making the findings of this study even more critical to consider. To best understand the refugee population in the United States exhaustively, replicating this needs assessment to evaluate other refugee groups is vital in developing a thorough repertoire of understanding for these integral populations of our community.

## Data Availability

All data generated or analyzed during this study are included in this article.*

## References

[1] Key displacement and solutions trends in the first half of 2024. https://www.unhcr.org/us/mid-year-trends#:∼:text=Over122.6millionpeopleareforciblydisplacedglobally&text=BytheendofJune,%2Dandmiddle%2Dincomecountries (2024, accessed September 30, 2024).

[2] History of UNHCR. UNHCR. https://www.unhcr.org/about-unhcr/overview/history-unhcr (2025, accessed June 22, 2025).

[3] Refugee Admissions . U.S department of state Bureau of Population, Refugees, and Migration Refugee Admissions. https://2009-2017.state.gov/j/prm/ra/, 2017.

[4] Cramer C Goodhand J . Try Again, fail again, fail Better? War, the state, and the ‘post–conflict’ challenge in Afghanistan. Dev Change 2002; 33(5): 885–909. DOI: 10.1111/1467-7660.t01-1-00253.

[5] The U.S. War in Afghanistan. Council on Foreign Relations. https://www.cfr.org/timeline/us-war-afghanistan

[6] Afghanistan Refugee Crisis Explained . USA for UNHCR (The UN Refugee Agency). https://www.unrefugees.org/news/afghanistan-refugee-crisis-explained/#:∼:text=AccordingtoUNHCR's2023Global,RepublicofIranandPakistan (2024, accessed 1 October 2024).

[7] Afghan immigrants in the United States. Migration Policy Institute. https://www.migrationpolicy.org/article/afghan-immigrants-united-states-2022#:∼:text=Between2010and2022%2Cthe,populationgrewby16percent (2024, accessed October 1, 2024).

[8] With the second anniversary of the Fall of Kabul approaching, thousands of Afghan refugees remain stuck in legal limbo. The Texas Standard.

[9] The Vietnamese community in Texas: history and impact. Texas State Historical Association. June 14, 2025. https://www.tshaonline.org/handbook/entries/vietnamese

[10] New profile reveals contributions of immigrant population to Houston metro area, its growing diversity and challenges. Migration Policy Institute. https://www.migrationpolicy.org/news/houston-report-2023 (2023, accessed October 2, 2024).

[11] Villarreal A . Where Texas goes, the nation follows: state legislatures and immigration enforcement. National Immigration Forum , https://immigrationforum.org/article/where-texas-goes-the-nation-follows-republican-controlled-state-legislatures-and-immigration-enforcement/ (2024, accessed June 14, 2025).

[12] Charles Perry . Texas Senate Bill 4. https://capitol.texas.gov/tlodocs/85R/billtext/pdf/SB00004F.pdf (2016, accessed June 14, 2025).

[13] Potter D Dawson L DeLisi A , et al. The 44th Kinder Houston area survey: Destination Houston: A growing region’s path to prosperity. Rice University Kinder Institute for Urban Research, 2025. DOI: 10.25611/XKN2-AT90.

[14] Mann EM Klosovsky A Yen C , et al. Health challenges in refugee resettlement: an innovative multi-sector partnership to improve the continuum of care for resettled refugees. J Trav Med 2020; 27(7): taaa103. DOI: 10.1093/jtm/taaa103. PMC7649377 32577767

[15] Pace M Al-Obaydi S Nourian M , et al. Health services for refugees in the United States: policies and recommendations. Publ Pol Adm Res 2015; 5.

[16] Matsangos M Ziaka L Exadaktylos AK , et al. Health status of Afghan refugees in Europe: policy and practice implications for an optimised healthcare. IJERPH 2022; 19(15): 9157. DOI: 10.3390/ijerph19159157. 35954518 PMC9368211

[17] Azizi N Delgoshaei B Aryankhesal A . Barriers and facilitators of providing primary health care to Afghan refugees: a qualitative study from the perspective of health care providers. Mjiri 2021; 35: 1. 33996652 10.47176/mjiri.35.1 PMC8111621

[18] Alemi Q James S Cruz R , et al. Psychological distress in Afghan refugees: a mixed-method systematic review. J Immigr Minority Health 2014; 16(6): 1247–1261. 10.1007/s10903-013-9861-1 PMC3912229 23784146

[19] Kerwin D . The US refugee resettlement program — a return to first principles: how refugees help to define, strengthen, and revitalize the United States. Journal on Migration and Human Security 2018; 6(3): 205–225.

[20] Benson GEK . Comparing refugee resettlement services: a new global dataset and typology. Refug Surv Q 2025; 44: 12–36.

[21] Wright J Williams R Wilkinson JR . Health needs assessment: development and importance of health needs assessment. BMJ 1998; 316(7140): 1310–1313. 9554906 10.1136/bmj.316.7140.1310 PMC1113037

[22] Hollifield M Verbillis-Kolp S Farmer B , et al. The Refugee Health Screener-15 (RHS-15): development and validation of an instrument for anxiety, depression, and PTSD in refugees. Gen Hosp Psychiatry 2013; 35(2): 202–209. 23347455 10.1016/j.genhosppsych.2012.12.002

[23] PARPARE screening tool. https://prapare.org/the-prapare-screening-tool/

[24] Rek SV Bühner M Reinhard MA , et al. The COVID-19 Pandemic Mental Health Questionnaire (CoPaQ): psychometric evaluation and compliance with countermeasures in psychiatric inpatients and non-clinical individuals. BMC Psychiatry 2021; 21(1): 426. 34465319 10.1186/s12888-021-03425-6 PMC8406012

[25] Lipson JG Miller S . Changing roles of afghan refugee women in the United States. Health Care Women Int 1994; 15(3): 171–180. 8002414 10.1080/07399339409516110

[26] Van Der Heide I Wang J Droomers M , et al. The relationship between health, education, and health literacy: results from the Dutch adult literacy and life skills survey. J Health Commun 2013; 18(sup1): 172–184. 24093354 10.1080/10810730.2013.825668 PMC3814618

[27] Sharifian F Sadeghpour M Barton S , et al. English language learning barriers of Afghan refugee women in Australia. Int J App Linguistics 2021; 31(1): 65–78. DOI: 10.1111/ijal.12320.

[28] Field Listing - Total Fertility Rate . Central intelligance agency. https://www.cia.gov/the-world-factbook/field/total-fertility-rate/ (Accessed November 16, 2024).

[29] Kaplan J Lazarescou N Huang S , et al. Overview of challenges faced by refugees following resettlement in Houston, Texas: a qualitative study at five refugee resettlement agencies. IJMHSC 2022; 18(1): 1–15.

[30] Shahawy S Onwuzurike C Premkumar A , et al. Perspectives of women of refugee background on healthcare needs in a major urban metropolitan community in the US : a qualitative needs assessment. Health Soc Care Community 2022; 30(6): e5637–e5646. DOI: 10.1111/hsc.13989. 36111793 PMC10286114

[31] Ji X Chow E Abdelhamid K , et al. Utility of mobile technology in medical interpretation: a literature review of current practices. Patient Educ Couns 2021; 104(9): 2137–2145. 33653659 10.1016/j.pec.2021.02.019

[32] Mangrio E Sjögren Forss K . Refugees’ experiences of healthcare in the host country: a scoping review. BMC Health Serv Res 2017; 17(1): 814. 29216876 10.1186/s12913-017-2731-0 PMC5721651

[33] Mutitu A Zabler B Holt JM . Refugees’ perceptions of primary care: what makes a good doctor’s visit? Patient Experience Journal 2019; 6(3): 33–41. DOI: 10.35680/2372-0247.1382.

[34] Brickhill-Atkinson M Hauck FR . Impact of COVID-19 on resettled refugees. Prim Care 2021; 48(1): 57–66. 33516424 10.1016/j.pop.2020.10.001 PMC7538065

